# To Speak Up or Not to Speak Up, Organisational and Individual Antecedents That Undergird This Behaviour in Resource Constrained Region

**DOI:** 10.1111/jan.16446

**Published:** 2024-09-04

**Authors:** Kwadwo Asante

**Affiliations:** ^1^ Tomas Bata University in Zlin Zlin Czech Republic

**Keywords:** belief, fussy‐set qualitative comparative analysis, Ghana, healthcare workers, norm, perceived organisational support, psychologically safe environment, speaking up, value

## Abstract

**Aim:**

To examine the organisational (i.e., perceived organisational support and psychologically safe environment) and individual (i.e., value, belief and norm) antecedents that strengthen healthcare workers' speaking‐up behaviour in a developing economy.

**Design:**

The study uses a cross‐sectional design to gather the same data from healthcare workers within the Ashanti Region of Ghana.

**Methods:**

The data collection happened between 15 June and 30 August 2023. A sample of 380 healthcare workers was selected from 20 facilities in the Ashanti Region of Ghana. A configurational approach, a fussy‐set qualitative comparative analysis, was used to identify the configurations that caused high and low speaking‐up behaviour among the study sample.

**Results:**

The study results reveal that whereas four configurations generate high speaking‐up behaviour, three configurations, by contrast, produce low speaking‐up behaviour among healthcare workers.

**Conclusion:**

Results suggest that in so far as organisational support systems which take the form of a psychologically safe environment and perceived organisational support are vital in relaxing the hierarchical boundaries in a healthcare setting to improve healthcare workers' speaking‐up behaviour, the individual value‐based factors that take the form of values, beliefs and norms are indispensable as it provides the healthcare workers with the necessary inner drive to regard speaking‐up behaviour on patient safety and care as a moral duty.

**Impact:**

Healthcare workers' speaking‐up behaviour is better achieved when organisational support systems complement the individual norms, values and beliefs of the individual.

**Reporting Method:**

Adhered to Strengthening Reporting of Observational Studies in Epidemiology guidelines.

**Patient or Public Contribution:**

No patient or public contribution.

## 1 Introduction

The severity of fatalities and accidents in the healthcare sector has made scholars compare it to other large‐scale hazardous industries, such as aviation, chemical and nuclear sectors, where upstream institutional lapses translate into deadly downstream catastrophes (Reason 1997). A more significant part of the healthcare sector's fatalities and accidents come from medical errors (MEs). Medical errors, which hereafter would be referred to as MEs inadvertently, have become an obstinate and considerable challenge affecting the quality of healthcare delivery in developed and developing economies. According to the Institute of Medicine, on average, every patient is exposed to at least one ME each day in a health facility (Aspden et al. 2007), making it the third primary source of death in the United States (Makary and Daniel 2016). A meta‐analysis study by Mekonnen et al. (2018) concluded that the ME rate in the African healthcare setting is relatively widespread, occurring in a median of 57.4% of all prescriptions made.

Whereas some studies indicate that 60%–70% of these MEs happen as a result of a communication mishap (Donaldson, Corrigan, and Kohn 2000; Joint Commission 2008), 23% of those errors occur precisely because of healthcare workers' reluctance to speak up (Peadon, Hurley, and Hutchinson 2020). Speaking up is raising concerns for the sole purpose of safeguarding a patient's safety or improving the quality of healthcare upon becoming aware of a danger or deviation of actions of others (Lyndon et al. 2012). Speaking up, therefore, constitutes a form of self‐confident pronouncement intended for corrective purposes, making it a cost‐effective way to lessen MEs (Morrison 2014).

Notwithstanding the broad recognition that healthcare workers need to speak up to make their concerns known or challenge specific actions when deemed necessary, recent studies indicate that healthcare workers still regard this act as a difficult endeavour and may be unwilling to challenge the status quo even when immediate action is required to protect patient life (Jackson 2022; Lee et al. 2022). Health workers' ability to speak up is more undermined in developing economies as the region's work culture highly reveres respect for authority and niceness (Lee and Quinn 2020), and those who seek to break this code can become the primary target of scrutiny and even investigation including punitive action (Jackson et al. 2013). Bearing in mind the dangers healthcare workers' silence may pose on patient safety and the quality of healthcare delivery, especially in resource‐constrained regions where healthcare facilities are overburdened with more cases and low logistical support (Martin and Armstrong 2020), the importance of healthcare workers speaking up cannot be understated particularly in resource‐constrained regions (Fagan, Parker, and Jackson 2016). Because many MEs are preventable, conditions facilitating speaking up among healthcare workers have dominated the discourse among academics and institution managers (Okuyama, Wagner, and Bijnen 2014). Therefore, understanding the organisation and individual factors that strengthen and weaken healthcare workers' speaking‐up behaviour in resource‐constrained regions is crucial for patient safety and effective healthcare delivery.

## 2 Background

Although the number of studies on speaking up across the healthcare sector has increased, the literature still has wide knowledge gaps that must be addressed (Blenkinsopp et al. 2019). First, looking at the hierarchical nature of the healthcare setting, healthcare workers may be reluctant to speak up on a breach of procedure, primarily when their views are likely to conflict with those in authority (Sur et al. 2016; Blenkinsopp et al. 2019). Second, the overt hierarchical nature of the healthcare sector makes it difficult for subordinates to challenge authority (Gladwell 2008). Third, the extant literature position that implementing initiatives such as developing healthcare workers' assertive communication skills and building their collective awareness about the kind of behaviours or events to voice out (McKenzie et al. 2019) may not always suffice when prioritising relationships with superiors or colleagues become the utmost concern of most healthcare workers' (Friedman, Hayter, and Everett 2015).

For instance, notwithstanding the implementation of the Freedom to Speak Up initiative and Speak Up Guardians in the English National Health Service (Adams et al. 2020), a report suggests that most healthcare workers are still reluctant to speak up (Violato 2022). Healthcare workers' enthusiasm to speak up about patient safety is complex and may move beyond organisation‐sponsored programmes (Noort, Reader, and Gillespie 2019). Despite these concerns, studies investigating individual value‐based factors on organisational speaking‐up interventions remain underexplored in the literature (Sur et al. 2016). Key central research questions remain unresolved, such as whether a person's inclination to act when they see a patient's care undermined and believe their actions can save the patient's life even when their activity will challenge their superiors is adequate in inspiring healthcare workers to speak up. Tackling this research question is crucial because the hierarchical arrangement within the healthcare sector could make healthcare workers remain silent even when they know that speaking up could save a patient's life (Lee et al. 2022).

Accordingly, to address the abovementioned gaps, the value–belief norm theory (VBN) (Stern et al. 1999) is an essential theoretical lens to explain the interplay that individual value‐based factors such as values, beliefs and norms have on their speaking‐up behaviour. The value belief norm posits that an individual is likely to take action when they value something, perceive it as threatened and believe their actions can restore its worth (Stern et al. 1999). The study, therefore, strengthens the theoretical predictors of speaking‐up behaviour in the healthcare literature by using value belief norms as its theoretical lens. Considering that speaking up may be more difficult in cultures such as Ghana, where respect for authority, niceness and hierarchical obligations are highly admired (Lee and Quinn 2020), the study theorised that the personal values, norms and beliefs of voicing out a concern when a wrong deed is committed could inspire a speaking‐up behaviour which is crucial in the health facility.

**FIGURE 1:**
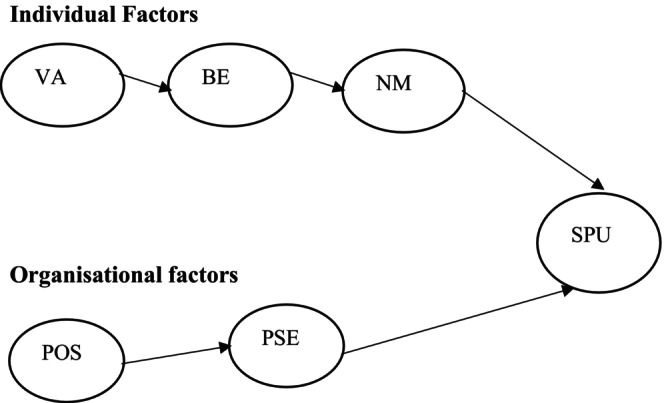
Conceptual model. BE, belief; NM, norms; POS, perceived organisational support; PSE, psychological safe environment; SPU, speaking‐up behaviour; VA, values.

Additionally, unlike developed economies, healthcare workers in developing countries such as Ghana operate in resource‐constrained facilities that are constantly overburdened with more cases (Martin and Armstrong 2020). Therefore, to strengthen healthcare workers' values, beliefs and norms in such a working environment, a higher‐level organisational support mechanism has to exist to counteract the negative consequence of these poor working conditions on individual values (Riggle, Edmondson, and Hansen 2009). Drawing on the perceived organisational support theory, the study theorised that positive perceived organisational support and a psychologically safe environment would minimise healthcare workers' job stressors and could strengthen the individual valued‐based antecedents' on their speaking‐up behaviour. Lastly, looking at the limitation of often used symmetrical analytical approaches in speaking‐up studies, the study used a configurational approach, a fussy‐set qualitative comparative analysis (fsQCA), in response to calls for new healthcare studies to adopt a more robust analytical method (Asante and Novak 2023). The configurational analysis produces a more fine‐grained understanding of the model relationship, providing the outcomes to achieve ideal managerial decisions. Against this backdrop, the study's main objective is to explore the organisational (i.e., perceived organisational support and psychologically safe environment) and individual (i.e., value, belief and norm) antecedents that strengthen healthcare workers' speaking‐up behaviour in a developing economy, Ghana. Figure 1 below summarises the model of the study.

## 3 Study

### 3.1 Aims

The study's main was to investigate the organisational (i.e., perceived organisational support and psychologically safe environment) and individual (i.e., value, belief and norm) antecedents that strengthen healthcare workers' speaking‐up behaviour.

### 3.2 Design

The study uses a cross‐sectional design to gather the same data from healthcare workers within the Ashanti Region of Ghana (Bryman 2016). The Strengthening Reporting of Observational Studies in Epidemiology guided the study (Von Elm et al. 2014).

### 3.3 Sample/Participants

According to the World Health Organisation report, Ghana is one of the few countries in Africa with more than 100,000 healthcare workers (World Health Organization 2021). The country's healthcare system is unevenly distributed, with the urban centres having the highest occupancy rate of hospitals, clinics, healthcare workers and pharmacies. Because the Ashanti Region of Ghana has one of the country's highest numbers of healthcare facilities and workers, the region was used as the study context. The region has a total of 1654 health facilities, with 1120 as community health planning services (CHPS), 29 clinics, 71 maternity homes, 165 health centres, 5 polyclinics, 26 district hospitals, 125 classified as other hospitals, 1 regional hospital, 1 university hospital and 1 teaching hospital (Ghana Health Service 2023). Therefore, using the healthcare workers in the region will improve the generalisability of the results to other regions of Ghana because they are well represented along the lines of primary, secondary and tertiary facilities. Following the recommendation of extant literature, when the available resources restrict a study's access to the entire population, purposive sampling becomes the most feasible sampling approach to use (Amarasena et al. 2015; Bryman 2016). Accordingly, the study purposely selected 20 healthcare facilities. Of the 20 selected facilities, 5 were clinics, 3 were maternity homes, 5 were district hospitals, 4 were polyclinics and 2 were other hospitals, and the region's teaching hospital was included because it remained the final referral point in the region. The study subsequently targeted healthcare workers employed in these 20 healthcare facilities.

However, to determine the sample size required to produce a suitable effect size, a priori power analysis was used following the threshold of a medium effect size of 0.15, a power of 0.95 and an alpha value of 0.05 (Memon et al. 2020). Since the proposed model had five predictors, a sample size of 138 became the requisite to achieve the medium effect size of 0.15. The required sample size of 138 was way below the sampled healthcare workers, 380. The inclusion criteria were full‐time healthcare workers with the necessary licence from the designated bodies: Ghana Medical and Dental Council, Nursing and Midwifery Council of Ghana, Allied Health Professions Councils and the Association of Health Service Administrators, Ghana (AHSAG). A selected sample was also required to have at least 1 year of postqualification working experience during the study. All employees who met these selection criteria were included in the sampling frame in a health facility.

After obtaining ethical clearance from the designated bodies, letters of introduction were sent to hospital administrators and medical superintendents describing the study's purpose and subsequently seeking their approval. After the approvals had been granted, meetings were held with departmental heads from medical, nursing, allied health and clinical services, pharmacy, dispensary and support services. The briefing offered departmental heads the opportunity to ask questions and seek clarity on the procedure for data collection and sample selection. Afterwards, a short debriefing was held with all the participants who met the selection criteria, explaining to them the purpose of the study and the benefits of the findings to the health sector. The healthcare workers were assured of confidentiality and again permitted to terminate participation anytime they felt uncomfortable continuing the study. Six hundred healthcare workers across the 20 healthcare facilities who met the selection criteria formed the sampling frame, of whom 380 were randomly selected. The questionnaires were sent to these individuals in person which must be completed during their off days or breaks. A total of 288 valid responses were obtained, giving a response rate of 75.5%. The data collection happened between 15 June and 30 August 2023.

### 3.4 Measures

The study adapted its measuring instruments from existing validated scales. Aside from the demographic scale, all the items were assessed on a 5‐point Likert scale (1, strongly disagree to 5, strongly agree). The study adopted the value belief norm scale from Batool et al. (2023) with three subconstructs: values on speaking up, beliefs on speaking up and norms on speaking up. However, the scale was slightly adjusted to suit the context of the study without limiting its conceptual meaning. The reported Cronbach alpha range of 0.751–0.957 for the three value belief norm constructs is consistent with what Kim et al. (2022) reported in their study >0.7 (see Table 2).

Healthcare workers' perceived organisational support was assessed using Eisenberger et al.'s (1986) eight‐item scale. These items were slightly modified to capture healthcare workers' perception of how their hospitals/departments have instituted the relevant support systems to ease their speaking up on MEs and patient safety. The perceived organisational support scale has recorded high internal consistency in various behavioural studies with often reported alpha values >0.8 (Asante and Novak 2023). Obtaining an alpha value of >0.7 on this study's perceived organisational support scale, as reported in Table 2, confirms the items' internal consistency.

The psychologically safe environment was assessed with five items adapted from the studies of Edmondson (1999) and Pavithra et al. (2022). The alpha value recorded on the psychologically safe environment scale is in tandem with the score reported in the study of Edmondson (1999) (i.e., α >0.8). Finally, four items were adapted from the Van Dyne, Ang, and Botero (2003) study to assess the healthcare workers' speaking‐up behaviour. The speaking‐up behaviour scale based on Van Dyne, Ang, and Botero (2003) study has been used within several fields to assess individual speaking‐up behaviours. In this study, speaking‐up behaviour obtained an alpha score of 0.9, confirming the scale's high reliability, as reported in the study of Gkorezis and Theodorou (2016).

### 3.5 Common Method Bias

Procedural and statistical methods were employed to address the issue of common method bias (CMB). With the procedural measure, the items were randomised to reduce the possibility of respondents guessing the relationship between the variables. Additionally, with the statistical method, the highly suggested Harman single‐factor test was estimated (Podsakoff et al. 2003). The total difference explained by a single‐factor component stood at 40.8%, confirming that common method bias was not a significant issue to be worried about in this study (Malhotra et al. 2017).

### 3.6 Data Analysis

As highlighted earlier, considering the constraint of the widely used symmetrical methods in speaking‐up studies, a configurational technique, fsQCA, was used. The fsQCA assesses alternative models by joining several factors (i.e., causal recipes) to predict an outcome (Pappas and Woodside 2021). The fsQCA encompasses data calibration, truth tabulation and counterfactual analyses (Olya et al. 2020). First, at the data calibration, guided by the recommendations of the extant literature, the data were calibrated into three sets of conditions: full membership, crossover point and full nonmembership (Ragin 2008). The study used the percentile function in SPSS 28.0 (Frequencies > Statistics > Percentile) to calibrate the data into the three anchors (fuzzy scores = 0.95, 0.50 and 0.05) to identify the degree of membership for each construct (Pappas and Woodside 2021). With the second phase of the fsQCA, the truth table contains a list of all possible conditions that determine an outcome (i.e., healthcare workers' speaking‐up behaviour). The truth table was estimated with the frequency and consistency set at 3 and 0.8, respectively (Pappas and Woodside 2021). The proportional reduction in inconsistency (PRI) in the truth table becomes very useful in social science research as it prevents the simultaneous repetition of a subgroup of solutions in both the presence and negation of an outcome (Greckhamer et al. 2018; Pappas and Woodside 2021). According to Greckhamer et al. (2018), the PRI should be high and close to raw consistency scores, and the score for reported configurations should be >0.5. Guided by this criterion, three configurations failed this test (see Table 1) and were deleted from the truth table (Ragin 2008). Table 1 shows the truth table for the model outcomes.

**TABLE 1:** Truth table.

VA	NOM	BE	PSE	POS	Number	SPU	Raw consist.	PRI consist.	SYM consist
1	0	0	1	1	8	1	0.982324	0.772412	0.772413
1	1	0	1	1	12	1	0.974674	0.848812	0.848812
1	1	1	1	1	20	1	0.970046	0.864986	0.922468
1	1	0	1	0	12	1	0.966455	0.720755	0.7318
0	1	1	0	0	12	1	0.95509	0.583332	0.583333
0	1	0	1	1	8	1	0.943583	0.607756	0.607756
1	1	0	0	1	16	1	0.93645	0.628206	0.628205

Abbreviations: BE, belief; NM, norms; POS, psychological, organisational support; PRI consist., proportional reduction in inconsistency; PSE, perceived safety environment; raw consist., raw consistency; SPU, speaking‐up behaviour; SYM consist, symmetric consistency; VA, values.

## 4 Results

### 4.1 Confirmatory Factor Analysis (CFA) and Reliability and Validity Analysis

The traditional measurement criteria, such as convergent, discriminant and construct validity, were used (Hair et al. 2019). The study used partial least square structural equation modelling (Smart‐PLS) 4.1 to assess the model's validity and reliability (Ringle, Wende, and Becker 2024). On the assessment of the constructs' convergent validity, results in Table 2 confirm that the scales' average variance extract (AVE) and factor loadings were within the acceptable threshold of >0.6 for factor loadings and 0.5 for AVE (Hair et al. 2019). Except for two speaking‐up items, two psychologically safe environment items, one perceived organisational support item and all the other items, factor loadings surpassed the suggested threshold of 0.7. Guided by the recommendations of Malhotra and Dash (2011), these items were deleted because their inclusion affected the scales' validity and reliability. The composite reliability (CR) criteria measured the constructs' reliabilities. According to Hair et al. (2019), a scale achieves high reliability when its CR >0.8 (Table 2). Per this criterion, the scales CR were above this threshold. With the assessment of the scales' discriminant validity, the heterotrait–monotrait (HTMT)—recommended to provide a more robust discriminant validity test than the widely used Fornell–Larcker criterion and cross‐loadings—is used (Henseler, Ringle, and Sarstedt 2015). According to Henseler, Ringle, and Sarstedt (2015), to achieve a stricter or more lenient threshold, HTMT should be lower than 0.85 or 0.95, respectively. In this study, all the constructs HTMT were lower than 0.90 (Table 3), confirming that discriminant validity is not an issue (Henseler, Ringle, and Sarstedt 2015). Finally, the standardised root‐mean‐square residual (SRMR) establishes the model's overall fitness (Henseler, Ringle, and Sarstedt 2015). Guided by the recommendations of Hu and Bentler (1999), a model achieves an acceptable fitness when its SRMR value is <0.080. The SRMR recorded a value of 0.078 with a Chi‐square (χ^2^) = 1092.176, indicating that the proposed model is fit for producing empirical evidence (Hu and Bentler 1999).

**TABLE 2:** Constructs of indicators and measurements.

Constructs	α	AVE	CR (rho_a)	Factor loading	Mean	SD
VA	**0.951**	**0.873**	**0.959**			
Working for the welfare of patients in our department is central.				0.957	4.14	1.045
I care for the sick and others in our department/hospital.				0.947	4.25	1.099
Preventing medical errors				0.944	4.35	0.977
Protecting patient safety is critical in the healthcare delivery process.				0.887	4.16	1.122
NM	**0.916**	**0.856**	**0.928**			
I feel obligated to speak up when a patient's safety is undermined.				0.910	3.98	1.027
My moral duty is to use all the appropriate channels to speak up if a protocol is broken.				0.937	4.07	0.971
Patient safety and the quality of healthcare delivery should be paramount.				0.928	4.18	0.965
BE	**0.771**	**0.681**	**0.806**			
When health workers break a rule or fail to adhere to standardised protocols, it often produces disastrous consequences for the patient.				0.885	3.82	1.070
Patients have as much right as health workers to exist.				0.835	4.01	1.064
If things continue on their present course in our health facilities, we will soon experience a significant breakdown in the quality of our healthcare delivery.				0.751	3.34	0.971
PSE	**0.838**	**0.753**	**0.859**			
I feel comfortable speaking‐up or reporting unprofessional behaviour.				0.883	3.58	1.160
I am confident I would receive support from my supervisor if I reported unprofessional behaviour.				0.847	3.53	1.147
People on my team/department sometimes reject others for voicing their concerns.				0.874	3.26	1.257
SPU	**0.846**	**0.866**	**0.849**			
I speak up and encourage others to get involved in patient safety issues.				0.936	4.02	0.937
I speak up when I have an idea for improving patient safety in my department.				0.926	4.02	0.863
I keep quiet instead of raising concerns when I see my superior making a poor clinical judgement.					2.36	1.370
I speak up when my colleague breaks a rule in my department.					3.70	1.161
POS	**0.921**	**0.765**	**0.937**			
The hospital I worked with has given me the skills to speak up effectively if I experience unprofessional behaviour.				0.934	3.58	1.095
I have the skills to speak up effectively if others experience unprofessional behaviour.				0.842	3.64	1.076
The department/hospital I work with is interested in my opinions on our healthcare delivery process.				0.876	3.48	1.282
The hospital/department I work with values my concerns about patient safety.				0.910	3.78	1.153
The hospital/department I work with takes pride in my voice.				0.712	3.21	1.325

*Note:* Bolded are the reliability and validity checks. Unbolded are central tendency measures.

Abbreviations: AVE, average variance extract; BE, belief; CR, composite reliability; NM, norms; POS, psychological organisational support; PSE, perceived safety environment; SD, standard deviation; SPU, speaking‐up behaviour; VA, values; α, Cronbach alpha.

**TABLE 3:** Heterotrait–monotrait ratio.

	BE	NM	POS	PSE	SPU	VA
BE						
NM	0.828					
POS	0.582	0.520				
PSE	0.537	0.475	0.842			
SPU	0.792	0.782	0.845	0.802		
VA	0.719	0.779	0.373	0.205	0.496	

Abbreviations: BE, belief; NM, norms; POS, perceived organisational support; PSE, psychological safe environment; SPU, speaking‐up behaviour; VA, values.

### 4.2 Sample Description

The results in Table 4 show that the healthcare workers had a mean age of 26.5, with 180 (62.5%) being females. Additionally, a significant part of the respondents either have a diploma in nursing education (31.6%) or a bachelor's degree as their highest level of education. The results are consistent with the recent upgrade in Ghanaian nursing education as diploma certification becomes the minimum entry requirement to the nursing profession. The respondents are engaged in varied work roles as healthcare professionals, with the largest, 36.5%, involved in nursing. With descriptive statistics of the primary constructs (see Table 2), the item mean values were from 2.36 to 4.35, suggesting a disagreed‐to‐agreed rating.

**TABLE 4:** Characteristics of study respondents.

Demographic variable	Category	Frequency	(%)
Gender	Male	108	37.5
	Female	180	62.5
Age	25–35 years	180	62.5
	36–45 years	81	28.1
	Above 45 years	27	9.4
Level of education	Up to nurse assistant	35	12.2
	Diploma in Nursing	91	31.6
	Bachelor's degree	115	39.9
	Master's/PhD	47	16.3
Job status/position	Medical	61	21.2
	Nursing	105	36.5
	Allied health	94	32.6
	Management and administration	28	9.7
Number of years they have worked as a healthcare worker	1–5 years	126	43.8
	6–10 years	81	28.1
	More than 10 years	81	28.1

### 4.3 Configurational Results

Table 5 indicates the sufficient configurations (i.e., recipes) with acceptable consistency set at >0.8 and coverage set at >0.3 to predict healthcare workers' speaking‐up behaviour. The fsQCA shows that healthcare workers' speaking‐up behaviour came from four configurations. The first configuration suggests that value, perceived organisational support and absence of belief lead to high speaking up among healthcare workers. The second configuration shows that high speaking‐up behaviour emerges from value, a psychologically safe environment, perceived organisational support and an absence of belief. The third recipe suggests that the presence of norm, a psychologically safe environment, perceived organisational support and the negation of belief increase healthcare workers' speaking‐up behaviour, particularly if they have an idea for improving patient safety and healthcare. The fourth configuration reveals that a high presence of value, norm and psychologically safe environment combined with perceived organisational support facilitates high speaking‐up behaviour among healthcare workers.

**TABLE 5:** Sufficient configurations towards HCWs' speaking‐up behaviour.

Configurations	Raw coverage	Unique coverage	Consistency
Configurations for HCWs' speaking‐up behaviour SPU = f (VA, NM, BE, PSE, POS)			
VA*~BE*POS	0.620	0.042	0.902
VA*NM*~BE*PSE	0.551	0.010	0.953
NM*~BE*PSE*POS	0.561	0.029	0.948
VA*NM*PSE*POS	0.619	0.092	0.956
Solution coverage: 0.834			
Solution consistency: 0.863			
Configurations for the absence of HCWs' speaking‐up behaviour ~SPU = f (VA, NM, BE, PSE, POS)			
~NM*~BE*~PSE*~POS	0.540	0.188	0.957
VA*~NM*~BE*POS	0.447	0.071	0.940
~VA*NM*BE~PSE*~POS	0.263	0.003	0.937
Solution coverage: 0.830			
Solution consistency: 0.876			

Abbreviations: *, Logical conjunction; ~, negation or absence; BE, belief; HCW, healthcare workers; NM, norms; POS, perceived organisational support; PSE, psychological safe environment; SPU, speaking‐up behaviour; VA, values.

Unlike symmetrical approaches, which cannot identify the configurations restricting healthcare workers' speaking‐up behaviour, the fsQCA identified three recipes that cause low speaking‐up behaviour among healthcare workers. Configuration 1 reveals that healthcare workers' decision not to speak up emanates from their lower levels of norm and belief, weak psychologically safe environment and absence of perceived organisational support. The results imply that healthcare workers with feeble personal norms and beliefs in patient safety and care and working under poor organisational support and psychologically safe environments are less likely to speak up to authority when a rule or procedure is broken. The second configuration suggests that the absence of norm, belief and the presence of perceived organisational support combined with value curtailed healthcare workers' speaking‐up behaviour. The third configuration indicates a low value and weak psychologically safe environment and perceived organisational support combined with norms and belief‐restricted healthcare workers' speaking‐up behaviour. The overall solution coverage for the conditions predicting healthcare workers speaking up was >0.80 (see Table 5), affirming the sufficiency of these configurations (Ragin 2008). The results from the fsQCA suggest that healthcare workers' decision to speak up or remain silent about patient safety is complex and may require institutional and individual factors to support its frequent occurrence.

Lastly, guided by Ragin's recommendation Ragin (2008), the necessary condition analysis (NCA) was computed to identify the condition required to influence healthcare workers' speaking‐up behaviour. Results in Table 6 indicate that none of the conditions can be categorised as necessary because the reported consistency values on the recipes were < 0.9 (Ragin 2008).

**TABLE 6:** Necessary conditions analysis for HCWs' speaking‐up behaviour.

	Consistency	Coverage
VA	0.861	0.695
~VA	0.433	0.631
NM	0.887	0.788
~NM	0.575	0.717
BE	0.714	0.906
~BE	0.782	0.687
PSE	0.794	0.834
~PSE	0.642	0.658
POS	0.841	0.768
~POS	0.573	0.689

Abbreviations: ~, negation or absence; BE, belief; HCW, healthcare workers; NM, norms; POS, perceived organisational support; PSE, psychological safety environment; SPU, speaking‐up behaviour; VA, values.

### 4.4 fsQCA Predictive Power

**FIGURE 2:**
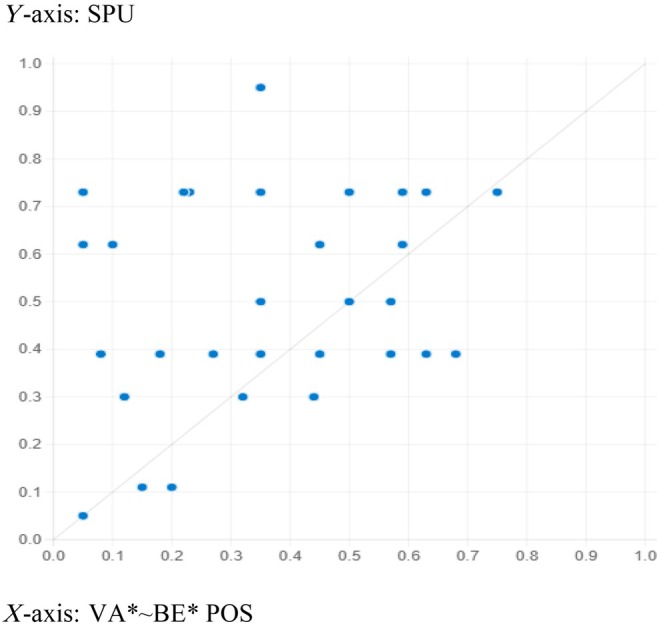
Configuration for *HCWs' speaking‐up behaviour* (VA* ~ BE*POS). Consistency: 0.901 and coverage: 0.620.

**FIGURE 3:**
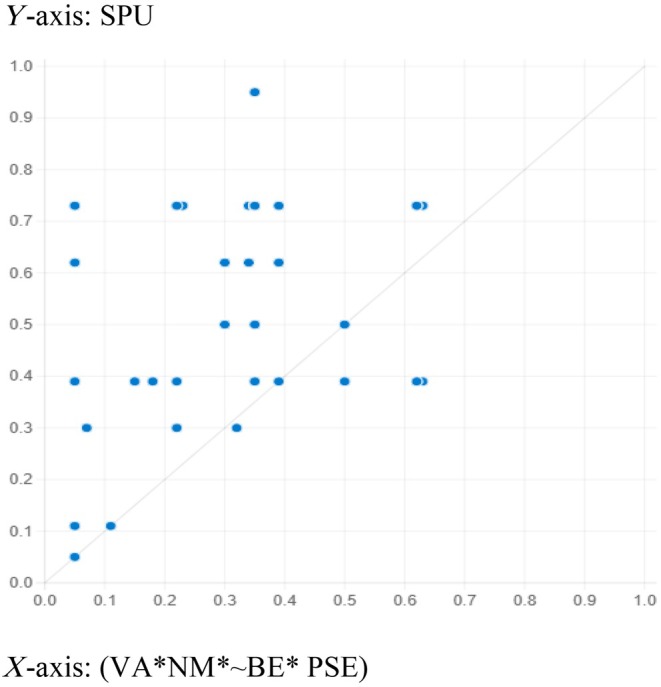
Configuration for *HCWs' speaking‐up behaviour* (VA*NM* ~ BE*PSE). Consistency: 0.958 and coverage: 0.559.

**FIGURE 4:**
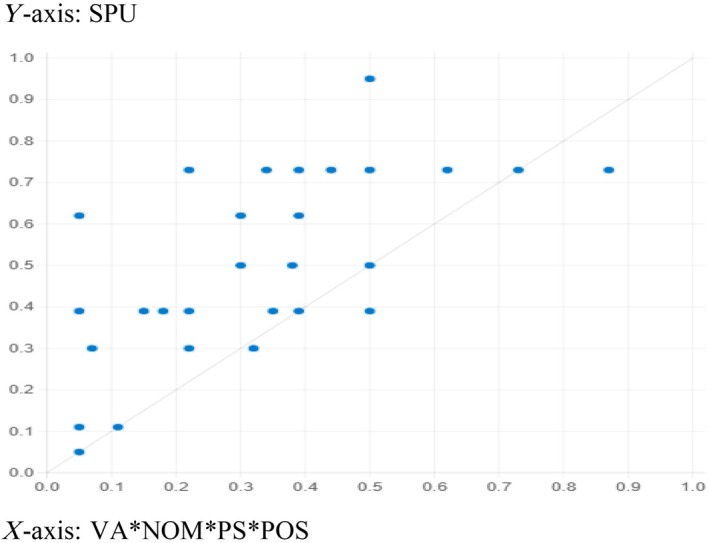
Configuration for *HCWs' speaking‐up behaviour* (VA*NOM*PS*POS). Consistency: 0.959 and coverage: 0.634. XY plots for sufficient recipes to predict HCWs' speaking‐up behaviour derived from subsample 2. BE, belief; HCW, Healthcare workers; NM, norms; POS, perceived organisational support; PSE, psychologically safe environment; SPU, speaking‐up behaviour; VA, values; *, logical conjunction; and ~, negation or absence.

The fsQCA solutions' predictability power was estimated to confirm the model's usability. For this purpose, the data were divided randomly into two subsamples. The first part of the subsample was employed to compute the sufficient outcomes by setting consistency and coverage at 0.8 and 0.3, respectively. Table 7 shows three adequate configurations generated from subsample one that predicted healthcare workers' speaking‐up behaviour. Next, subsample two was used to draw an XY plot and to estimate the consistency and coverage for the pull‐out recipes obtained from subsample 1. Figures 2, 3 and 4 show the results from the plot. The results generated from the plots affirm an acceptable consistency and coverage for all the sufficient configurations predicting healthcare workers' speaking‐up behaviour derived from subsample 2, supporting the recipes' explanatory power.

**TABLE 7:** Equifinality analysis.

Configurations	Raw coverage	Unique coverage	Consistency
Configurations for HCWs' speaking‐up behaviour SPU = f (VA, NM, BE, PSE, POS)			
VA*~BE*POS	0.602	0.038	0.901
VA*NM*~BE*PSE	0.529	0.010	0.959
VA*NM*PSE*POS	0.617	0.106	0.958
Solution coverage: 0.804			
Solution consistency: 0.874			

Abbreviations: *, Logical conjunction; ~, negation or absence; BE, belief; HCW, healthcare workers; NM, norms; POS, perceived organisational support; PSE, psychological safe environment; SPU, speaking‐up behaviour; VA, values.

## 5 Discussion

Notwithstanding the training and recognition that speaking up can reduce the harm caused to patients and save lives, healthcare workers do not continuously speak up when patient safety is undermined (Umoren et al. 2022). Often, most existing studies focused on the organisational factors that strengthen healthcare workers' speaking‐up behaviour. Studies investigating individual value‐based factors on organisational speaking‐up interventions remain untested in the literature. Considering the complexity of speaking‐up behaviour within the healthcare setting, the study examined the institutional (i.e., perceived organisational support and psychologically safe environment) and individual (i.e., value, belief and norm) antecedents that can increase the frequency of healthcare workers speaking‐up behaviour.

Results from the fsQCA showed that high speaking‐up behaviour among healthcare workers emerged from four configurations. The first configuration suggests that healthcare workers' readiness to speak up on patient safety and care emanates from their values and the high presence of perceived organisational support. As defined in the value belief norm theory, value becomes the desirable trans‐situational goal changing in importance and serves as a guiding signpost in a person's life (Stern et al. 1999). Personal values stemming from humanity, biospheric and self‐centred, significantly influence an individual attitude and behaviour (Kim et al. 2022). Healthcare workers who exhibit altruistic (humane) values, particularly towards the vulnerable (i.e., patients), will likely speak up for them when their healthcare is compromised or threatened.

The results affirm the findings of Kim et al. (2022) and Kiatkawsin and Han (2017), where personal values persuaded individuals to make decisions that benefited a vulnerable group. However, the findings from **configuration 1** imply that personal values have to combine with perceived organisational support to induce a sufficient influence on healthcare workers' speaking‐up behaviour. Therefore, for healthcare workers' values to facilitate the frequency of their speaking‐up behaviour, their perception of institutional support has to be high. Thus, healthcare workers' perception of how management cared about their work and respected their complaints becomes crucial to strengthening their values and affecting their speaking up. This result corroborates the conclusion of Landgren et al. (2016), where the perceptions of lack of support from management and instances of unheeded concerns thwarted healthcare workers' speaking‐up behaviour. The results have implications for healthcare managers. The results suggest that although the required support systems can be provided to healthcare workers, they can still be reluctant to voice their concerns when their values tend to be more self‐centred than those of the altruistic type. In the same way, high altruism may not be sufficient to produce a higher speaking‐up behaviour, especially when organisational support becomes nonexistent. This suggests that personal values and appropriate organisational support mechanisms must simultaneously be high to stimulate speaking up when needed.

**Configuration 2** revealed that personal values, norms and a psychologically safe environment influenced healthcare workers' tendency to speak up. Individual norms in this context reflect the beliefs that every healthcare worker has a moral responsibility to safeguard patient safety and care (Stern et al. 1999). The results indicate that the conviction in seeing the protection of patients as a moral duty coupled with a humane value (i.e., caring for the sick) and a high presence of a psychologically safe environment reinforced healthcare workers' speaking‐up behaviour. Since the hierarchical structure in a healthcare setting remains critical and facilitates the delivery and transfer of accumulated wisdom from a senior to a junior, it can discourage a subordinate from speaking to superiors about improper conduct (Belyansky et al. 2011). Therefore, if a psychologically safe environment is not built to reduce the perception of retribution, public humiliation and marginalisation which come with speaking up to authority (Landgren et al. 2016), healthcare workers will remain reluctant to speak up (Umoren et al. 2022). The result is consistent with the reasoning of Edmondson et al. (2016), as they posit that people can ask questions, propose alternatives or even disagree with colleagues, particularly those in power when they realise that their views will not result in any adverse personal consequence. The fsQCA confirms the insufficiency of a psychologically safe environment in producing the expected effect on healthcare workers speaking up, as their norms and values have to equally inspire them to see speaking up for patient safety as a call to duty and the right thing to do for the patient. The result is insightful because a health institution can create a safe environment. Nevertheless, self‐centred employees may not see the essence of speaking up about patient safety, especially when it involves challenging their direct superiors. Results from the study confirm the findings of Kim et al. (2022) and Kiatkawsin and Han (2017), as individual values and norms influence their decision to make choices that provide positive value for humanity.

**Configuration 3** suggests that norms, a psychologically safe environment and perceived organisational support resulted in high speaking‐up behaviour among healthcare workers. The results indicate that providing a safe climate and institutional support mechanism may not be adequate to inspire the necessary speaking up, especially when the healthcare worker does not regard this act as a moral responsibility. The result is in tandem with the views of Stern et al. (1999), as they posit that individuals who espouse fixed values and believe those standards are threatened would accept the personal duty to protect them. Similarly, although healthcare worker norms may inspire speaking‐up behaviour as a moral duty, working under a porous working environment where healthcare workers are continuously overworked could counteract the impact such positive standards tend to have on personal decisions (Martin and Armstrong 2020). Therefore, when proper support systems are not built by management to send a positive signal to healthcare workers in developing economies that management cares about their well‐being, it is likely to increase their dissatisfaction towards their job and consequently infiltrate their norms and lessen their enthusiasm to speak up even when they perceive it as a right thing to do. This view concurs with the conclusion of Pavithra et al. (2022), as their work posited that the non‐material support that healthcare workers received from their hospitals through recognition, trust and care strengthens speaking‐up behaviours. The results provide an essential insight to healthcare institutions in developing and other economies, revealing that failure to institute proper support mechanisms for healthcare workers will affect their job satisfaction, weaken their norms and reduce their desire to speak up.

The fourth configuration reveals that value, norm, psychologically safe environment and perceived organisational support increased healthcare workers' speaking‐up behaviour. According to the value–belief–norm theory, since personal values and standards activate a person's awareness and acceptance of responsibility, they become the cornerstone of every behaviour (Stern et al. 1999). Accordingly, personal values that are not aligned with altruism, which is selflessness in caring for the helpless, can reduce healthcare workers' enthusiasm to speak up even when patient safety and care are inconsistent with their professional training. From the fsQCA analysis, although personal values and norms stimulate a person's awareness and acceptance of responsibility (Stern et al. 1999), they may not be adequate to increase the frequency of speaking up, especially when the environment supporting speaking up is intimidating and futile to the healthcare worker career growth. The study advances existing literature by demonstrating that the lack of proper institutional support mechanisms can negatively undermine a high‐spirited individual whose norms, values and beliefs underscore patient safety as a call to duty from speaking up when a patient's safety is medically undermined.

Far from symmetrical analysis, the fsQCA identified recipes that lead to low speaking‐up behaviour among healthcare workers. For instance, the results indicate that healthcare workers were less likely to speak up when they had weakened personal norms and beliefs about the importance of speaking up for patient safety and when the psychologically safe environment and management support tended to be nonexistent. Also, it was evident that the availability of organisational support and values and feeble norms and beliefs about the importance of speaking up for patient safety inhibited healthcare workers' speaking‐up behaviour. Finally, low speaking‐up behaviour among healthcare workers emerged from strong personal norms and beliefs, a low psychologically safe environment and organisational support. The fsQCA analysis confirms that using only symmetrical analysis for speaking‐up behaviour studies may not provide a complete picture of the conditions that facilitate high and low speaking‐up behaviours among healthcare workers, as reported in the extant literature (Sur et al. 2016; Blenkinsopp et al. 2019). The study results add more nuances to the extant literature by identifying the conditions that explain healthcare workers' speaking‐up behaviour. This result, therefore, confirms the significance of using asymmetrical methods in healthcare studies.

### 5.1 Strengths and Limitations

The study has several strengths. The first strength is that it examined the organisational (i.e., perceived organisational support and psychologically safe environment) and individual (i.e., value, belief and norm) antecedents that facilitate healthcare workers' speaking‐up behaviour in a constrained region. Secondly, it is the first to adopt an asymmetrical approach in the healthcare literature to understand the combination of recipes that increase and restrict speaking‐up behaviour among healthcare workers in a developing economy. Again, the study included various categories of healthcare workers in the study sample, thereby improving the generalisability of the conclusions to all clusters of healthcare workers. Although the results advance the literature about the conditions that strengthen healthcare workers' speaking‐up behaviour, they still have limitations. First, using a cross‐sectional design will restrict the study from making a more dependable inference about the conditions that improve healthcare workers' speaking‐up behaviour. Again, although procedural measures were employed to limit the occurrence of common method bias, it is still possible that gathering dependent and independent data from a single source may cause reverse causality. Consequently, future studies should adopt a longitudinal design to strengthen the causal–effect relationship among its variables.

Another limitation is that the study did not examine how a respondent's health facility status (i.e., primary, secondary and tertiary) influenced their speaking‐up behaviours. Further studies should explore this relationship to allow us to comprehensively understand how a health worker's institution's affiliation shapes their speaking‐up behaviour. Lastly, the study did not account for the respondents' characteristics (e.g., academic qualification, job position and years of working experience) as part of the configurational analysis. Accordingly, future studies should include health workers' profiles as part of the configurational analysis to understand their effect on their speaking‐up behaviour.

## 6 Conclusion

The study reveals that in so far as organisational support systems such as a psychologically safe environment and perceived organisational support are central in relaxing the hierarchical boundaries in a healthcare setting to improve healthcare workers speaking up, the individual value‐based attributes that take the form of values, beliefs and norms are indispensable as it provides the healthcare worker with the necessary inner drive to regard speaking‐up behaviour on patient safety and care as the right thing to do. These findings offer helpful insight by pointing out the importance of moving beyond institutional‐centred interventions and equally focusing on training that can build up positive individual values, norms and beliefs to solidify organisational‐based intervention on healthcare workers' speaking‐up behaviour.

## Ethics Statement

Before collecting data from the participating hospitals, ethical clearance was obtained from the Ethics Committee of the University of Education, Ghana (reference number: 230018: 15/02/2023). Informed written consent was also obtained from all participants.

## Conflicts of Interest

The authors declare no conflicts of interest.

### Peer Review

The peer review history for this article is available at https://www.webofscience.com/api/gateway/wos/peer‐review/10.1111/jan.16446.

## Data Availability

The datasets generated during and analysed during the current study are not publicly available due to privacy or ethical restrictions but are available from the corresponding author upon reasonable request.
